# Effectiveness of Progressive Muscle Relaxation, Deep Breathing, and Guided Imagery in Promoting Psychological and Physiological States of Relaxation

**DOI:** 10.1155/2021/5924040

**Published:** 2021-07-02

**Authors:** Loren Toussaint, Quang Anh Nguyen, Claire Roettger, Kiara Dixon, Martin Offenbächer, Niko Kohls, Jameson Hirsch, Fuschia Sirois

**Affiliations:** ^1^Luther College, Decorah, IA, USA; ^2^Neurofeedback Clinic of Northern Colorado, Fort Collins, CO, USA; ^3^Gasteiner Heilstollen Hospital, Bad Gastein, Böckstein, Austria; ^4^University of Munich, Munich, Germany; ^5^Coburg University, Coburg, Germany; ^6^East Tennessee State University, Johnson City, TN, USA; ^7^University of Sheffield, Sheffield, UK

## Abstract

Research suggests that multiple forms of relaxation training (e.g., progressive muscle relaxation, meditation, breathing exercises, visualization, and autogenics) can help individuals reduce stress, enhance relaxation states, and improve overall well-being. We examined three different, commonly used approaches to stress relaxation—progressive muscle relaxation, deep breathing, and guided imagery—and evaluated them in a head-to-head comparison against each other and a control condition. Sixty healthy undergraduate participants were randomized to one of the four conditions and completed 20 minutes of progressive muscle relaxation, deep breathing, or guided imagery training that was delivered by recorded audio instruction. Baseline and follow-up assessment of psychological relaxation states were completed. Physiological relaxation was also assessed continuously using measures of electrodermal activity and heart rate. Results showed that progressive muscle relaxation, deep breathing, and guided imagery all increased the state of relaxation for participants in those groups, compared to participants in the control group. In each case, the increase was statistically significant and although the groups did not differ on relaxation before training, all groups were significantly higher on relaxation after training, as compared to the control group. Progressive muscle relaxation and guided imagery showed an immediate linear trend toward physiological relaxation, compared to the control group, and the deep breathing group showed an immediate increase in physiological arousal followed quickly by a return to initial levels. Our results lend support to the body of research showing that stress relaxation training can be effective in improving relaxation states at both the psychological and physiological level. Future research could examine stress relaxation techniques in a similar manner using designs where multiple techniques can be compared in the same samples.

## 1. Introduction

The present investigation seeks to better understand relaxation strategies by examining the efficacy of three different relaxation approaches in bringing about both psychological and physiological relaxation. Extant literature demonstrates the effectiveness of relaxation strategies, such as deep breathing, guided imagery, meditation, progressive muscle relaxation, and many other methods [1], but rarely are these strategies examined side-by-side in an experiment to evaluate relative effectiveness in promoting relaxation. It remains the case that we know only that many of these techniques are effective in promoting relaxation, not which techniques are most effective in bringing about relaxation. It is critically important that we better understand the most effective methods for increasing relaxation and the underlying self-regulation competencies because the relaxation response is directly and inversely related to chronic stress difficulties and poor mental and physical health [2–4]. The current study explores differences in the impact of progressive muscle relaxation, deep breathing, and guided imagery, as compared to a control condition, on psychological and physiological relaxation states in an undergraduate student sample. We chose to examine deep breathing, guided imagery, and progressive muscle relaxation because these three methods can be easily taught and practiced using a standardized audio recording, they are known to be effective, and they can realize almost immediate benefits. While we expect that all three methods will elicit relaxation responses, differences among the techniques are less clear and will be carefully examined in this comparative effectiveness research. We begin with a brief review of these techniques and then summarize our present aims.

### 1.1. Progressive Muscle Relaxation

Progressive muscle relaxation (PMR) is an actively engaging relaxation technique developed by Edmund Jacobson in the 1920s [5]. PMR involves participants actively contracting muscles to create tension and progressively releasing this [6]. The routine is repeated until participants acquire complete relaxation. This technique utilizes the principles of neuronal “top-down” and “bottom-up” processing to achieve results [7]. In “top-down” processing, participants use areas higher in the nervous system like the cerebral cortex and the cerebellum to contract muscles and gradually release the tension. In “bottom-up” processing, the holding and releasing of bodily tension produce proprioceptive stimulation from peripheral muscles that ascends to the brain via the spinal cord and the brainstem. With both stimulatory passages activated, PMR provides participants with quick and immediate relief.

The effects of this technique have been widely demonstrated in numerous studies. For instance, PMR can be useful in reducing stress. Pv and Lobo selected a simple random sample of 30 first-year nursing students, measured stress levels before and after PMR, and found a significant reduction in stress [8]. In another study with nursing students, PMR alleviated test anxiety [6], In this study, 49 students were randomized to a PMR treatment or control group, and both groups completed the Sarason Anxiety Questionnaire at baseline and completion of the experiment. The PMR treatment group received four 30-minute sessions of PMR. Although there was no significant difference before and after the experiment in the control group on test anxiety, a significant reduction in test anxiety was found in the treatment group.

Not only does PMR demonstrate stress-alleviating effects, but also it exhibits a positive influence on depression and anxiety. In one study, PMR was administered twice a day for five days to patients who had coronary heart disease [9]. The Hospital Anxiety and Depression Scale [10] was used to measure anxiety and depression. The results showed that PMR had a positive effect on reducing depression and anxiety in these patients. In another study, 50 hospitalized cancer patients were randomized into an experimental PMR group and a control group [11]. Patients in the PMR group showed reductions in anxiety as measured by the Generalized Anxiety Questionnaire, whereas patients in the control group showed no such improvements.

### 1.2. Deep Breathing

Deep breathing, which is also known as diaphragmatic breathing, is a technique that is based on the notion that mind and body integration produces relaxation [12]. The technique requires participants to contract the diaphragm, slowly inhaling and exhaling. Deep breathing appears to amplify blood oxygen levels, massages the inner organs located in or close to the abdomen, and possibly stimulates the vagus nerve [13].

Deep breathing has been shown to have a positive impact on various factors like stress, anxiety, and negative affect in numerous studies. For instance, in an experiment conducted in China, 40 healthy participants were recruited to investigate the effects of deep breathing on attention, negative affect, and stress [14]. Participants were randomized into control and experimental groups, both of which were measured on the variables of interest including attention, affect, and cortisol before and after the eight weeks of treatment conducted for 30 minutes every other day. Findings showed that over the course of deep breathing treatment, as compared to controls, participants increased sustained attention and decreased negative affect and cortisol levels.

Deep breathing is useful with multiple patient populations. A study of 4,793 presurgical patients was designed to investigate the benefits of using deep breathing and aromatherapy to reduce preoperative anxiety [15]. After deep breathing along with lavender aromatherapy, approximately 40% of the patients demonstrated a decrease in anxiety. Additionally, deep breathing has been shown to exert a positive influence on certain chronic conditions. In one quasi-experimental study, 32 patients with type II diabetes mellitus were trained on deep breathing and compared to untrained controls [16]. Deep breathing resulted in a significant decrease in Hamilton Anxiety Rating Scale scores for deep breathing trained participants, whereas untrained controls did not show any decrease in anxiety.

### 1.3. Guided Imagery

Guided imagery is a method for treating stress and anxiety in which one replaces disturbing memories with positive mental imagery [17]. This involves instructional guidance that invokes sensory experiences and behavioral and physiological responses. Sensory and contextual engagement are a key focus of this technique. The instructional guidance and the strong focus on the engagement of participants help gain greater perceptual detail of the images generated which creates a more realistic mental representation during the relaxation exercise [18].

Research supports the effectiveness of guided imagery in reducing stress and anxiety. In one study conducted to evaluate the effects of guided imagery on intraoperative anxiety for patients undergoing abdominal surgery under spinal anesthesia, guided imagery techniques were shown to significantly reduce anxiety [19]. In another study investigating the effects of 20 minutes of guided imagery on preoperative anxiety, guided imagery was shown to significantly reduce anxiety and cortisol levels [20]. Yet another study showed that hospital nurses working during the COVID-19 pandemic who were trained in guided imagery, as compared to controls, showed significantly greater decreases in death anxiety [17]. Other studies show the use of guided imagery outside of the medical setting. One study evaluating the use of nature-versus-urban-based guided imagery as an intervention for anxiety found a significant decrease in state anxiety amongst adults imagining both urban and natural settings [18], and the effect was strongest for nature-based guided imagery. Another study using guided imagery to facilitate self-forgiveness showed significant increases in self-forgiveness scores following seven five-minute sessions of guided imagery [21].

### 1.4. Multiple Methods

Studies have also been conducted that either combine or compare the effects of multiple methods of relaxation with one another. One study combined guided imagery, progressive muscle relaxation, deep breathing, mindfulness, exercise, aromatherapy, and yoga for nursing students during the course of one academic year to test for changes in anxiety [22]. This was a mixed-methods study, but quantitative analyses did not reveal significant changes in anxiety. Another study investigated the differences between PMR, guided imagery, and diaphragmatic breathing techniques on the quality of life for elderly individuals diagnosed with breast or prostate cancer [23]. Results of this study showed significant improvement following 45 minutes of training in quality of life and physical functioning after either of the three techniques were used. A study of undergraduates compared five minutes of deep breathing and progressive muscle relaxation to each other and other relaxation techniques such as the adapted dive reflex and the use of a weighted lap object for their effects on anxiety [7]. All of the separate techniques tested showed significant reductions in anxiety, but deep breathing and progressive muscle relaxation techniques appeared to be responsible for the greatest amount of anxiety reduction. Another study compared the use of PMR and guided imagery and its effects on stress, anxiety, and depression for pregnant women [24]. Participants in this study participated in two twenty-minute sessions of progressive muscle relaxation and guided imagery exercises. The combination of these techniques resulted in decreased stress and depression, but not anxiety.

### 1.5. Present Study

The existing research has provided a good evidence base to support the usefulness of multiple forms of stress relaxation techniques for the reduction of stress, anxiety, and depression and the improvement of quality of life, relaxation states, and positive mental health. Often these studies have examined specific patient or demographic populations and used clinicians to train individuals in these techniques. Equally often, the outcomes of interest are stress, anxiety, and depression, whereas the most direct outcome of interest, that is the state of relaxation, is commonly missing. Furthermore, not only are relaxation states not commonly assessed, but also when they are physiological parameters are not included in assessment, leaving a comprehensive picture of the efficacy of these stress relaxation states incomplete. Consequently, we sought to design an experiment comparing three of the more common stress relaxation techniques—PMR, deep breathing, and guided imagery—in healthy young undergraduates. We also sought to directly assess stress relaxation states and physiological manifestations of relaxation across a 20-minute relaxation session guided by a scripted audio instructional program. We hypothesized, based on existing literature, that each of the tested techniques would lead to both distinguishable psychological and physiological states of relaxation, as compared to controls.

## 2. Method

### 2.1. Participants

Participants included 60 undergraduate students who participated for extra credit in an undergraduate psychology course. Meta-analyses of several types of stress relaxation techniques show pooled effect sizes of moderate-to-large magnitude [3]. Using *η*^2^ = .06 as a conservative estimate of effect size for the group by time interaction of our least powerful analysis, an alpha of .05, and required power of .90, 60 participants were required for this study. The majority were female (71.4%) and the average age was 19.64 (SD = 1.32; range = 18–23). Female/male proportions, *χ*^2^ = 4.54, *p*=0.21, and average age, *F* = .58, *p*=0.63, did not differ across the groups. Participants were recruited through an introductory psychology student pool. All participants provided informed consent prior to beginning the study, and the study was approved by the institutional ethics committee.

### 2.2. Measures

#### 2.2.1. Psychological Stress Relaxation

The Smith Relaxation States Inventory-3 [25] was used as a self-report assessment of the effectiveness of the relaxation exercises. The Smith Relaxation States Inventory-3 consists of 38 items measuring 18 relaxation states and 3 stress states (somatic stress, worry, and negative emotion). Researchers can conceptualize the relaxation states in 5 categories: basic relaxation (sleepiness, disengagement, physical relaxation, rested/refreshed, and mental relaxation), core mindfulness (mindful acceptance, mindful quietness, mindful centeredness, mindful awareness, mindful awakening, and mindful innocence), mindful doing (trust, energized, and happy), mindful giving (thankful and loving, prayerful), and deep mindfulness (awe and wonder, deep mystery, and timeless, boundless, infinite, at one). Example items are “My mind is silent and calm,” “My body is physically relaxed,” and “I feel at peace.” Participants are asked to rate each item according to how they feel “right now” on a 6-point Likert scale of 1 (*not at all*) to 6 (*maximum*). The Smith Relaxation States Inventory-3 possesses good reliability and validity. [25] In the present study, the total score was used and demonstrated excellent internal consistency at both baseline and follow-up assessment (*αs* > .90).

#### 2.2.2. Psychophysiological Stress Relaxation

Psychophysiological measures of autonomic nervous system arousal were collected using the Biopac MP35 lab software and hardware (https://www.biopac.com). Biopac hardware and software is considered a gold-standard psychophysiological data collection system by some [26] and has been used in 4,440 studies that measured electrodermal activity and 10,400 studies that measured electrocardiographic activity [27]. The Biopac MP35 measured the participant's phasic changes in electrodermal activity (i.e., skin conductance) and heart rate (HR). Electrodermal activity was assessed at the first and second fingers of the nondominant hand. Heart rate was assessed using a standard triple-lead configuration where sensors were placed above each ankle and on the wrist of the nondominant hand. Psychophysiological data were continuously monitored and reduced to five-minute segments where the average value for each five-minute segment was used for analysis.

#### 2.2.3. Controls

Age and sex (male/female) were collected as control variables.

### 2.3. Procedure

Sixty participants were randomly assigned to one of three stress relaxation groups (deep breathing, PMR, or guided imagery) or a control group. Participants arrived at the laboratory, were greeted by a researcher, and read and signed the informed consent. All sessions were conducted in the evening between 7:00 p.m. and 9:00 p.m., Monday through Friday, during the fall and spring semesters. Participants were asked to refrain from the consumption of alcohol, nicotine, and caffeine 12 hours prior to participation. Participants were connected to the Biopac MP35 hardware to begin monitoring baseline psychophysiological data and then completed the baseline relaxation self-report questionnaire. Participants then completed the appropriate 20-minute exercise for their assigned group. In the control group, participants were offered several popular news or sports magazines to read (e.g., TIME, Sports Illustrated). Participants in the PMR group learned how to tense and relax muscle groups to bring about a state of relaxation. Participants in the deep breathing group learned how to use their breathing to invoke relaxation by breathing deeply, slowly, and attending to their breath. Guided imagery participants learned how to use mental representations to bring about a state of relaxation. All relaxation exercises were taught by recorded verbal instructions that were developed and disseminated by Dr. Jonathon Smith [25]. Once the assigned exercise was completed, participants were disconnected from the psychophysiological recording equipment, completed the follow-up relaxation self-report questionnaire, were thanked for their participation, and were dismissed.

### 2.4. Analysis

Data were examined using descriptive statistics (means and standard deviations) and repeated measures analyses of variance. Physiological data was first reduced to 5-minute epochs and examined using repeated measures analyses of variance. Group by time interactions were the statistical test of interest, and in the presence of a group by time interaction, simple effects were examined. All analyses included age and sex (male/female) as control variables. Data met assumptions for statistical hypothesis testing, and statistical significance was set at *p* < 0.05.

## 3. Results

### 3.1. Psychological Results

**Figure 1:**
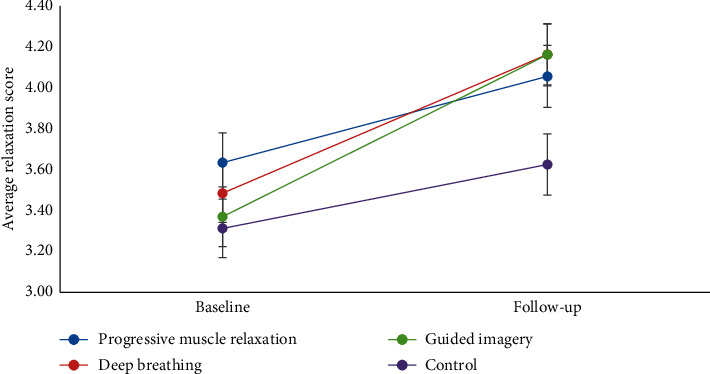
Baseline and follow-up levels of psychological relaxation states for participants in progressive muscle relaxation, deep breathing, guided imagery, and control groups. *Note.* Error bars represent standard errors.

Psychological changes in self-reported relaxation are displayed in Figure 1. Changes in levels of relaxation differed across the four groups, *F* = 2.81, *p*=0.048, *η*^2^ = .14. The control group (baseline to follow-up difference = 0.31, *p*=0.019) showed a slight increase in relaxation from baseline to follow-up assessment, but this change was considerably smaller than that of the relaxation groups. The PMR (baseline to follow-up difference = 0.42, *p*=0.002), deep breathing (baseline to follow-up difference = 0.68, *p* < 0.001), and guided imagery (baseline to follow-up difference = 0.79, *p* < 0.001) groups all showed statistically significant increases from baseline to follow-up assessment. While differences between the four groups were not statistically significant at the baseline (*ps* > 0.118), PMR (mean difference = 0.43, *p*=0.046), deep breathing (mean difference = 0.54, *p*=0.014), and guided imagery (mean difference = 0.54, *p*=0.015) groups were significantly higher on follow-up assessment relaxation scores compared to the control group. None of the relaxation groups differed from one another on follow-up assessment relaxation scores (*ps* > 0.621).

### 3.2. Physiological Results

**Figure 2:**
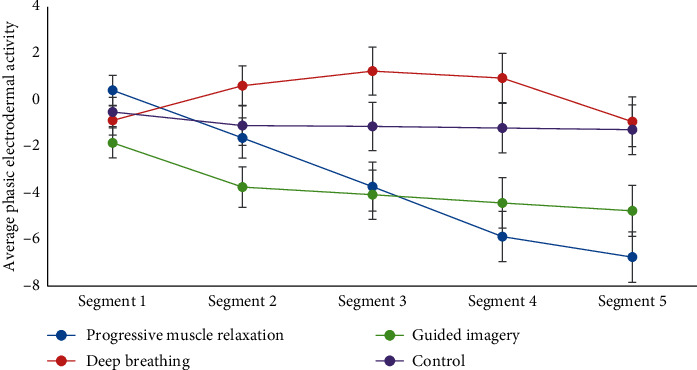
Changes in average phasic electrodermal activity (skin conductance) for participants in progressive muscle relaxation, deep breathing, guided imagery, and control groups. *Note.* Error bars represent standard errors. Segment 1 is a five-minute baseline.

Physiological relaxation evidenced by changes in electrodermal activity is displayed in Figure 2. Changes in levels of electrodermal activity differed across the four groups, *F* = 8.18, *p* < 0.001, *η*^2^ = 0.31. The control group, *F* = 0.36, *p*=0.837, *η*^2^ = 0.03, showed no changes in electrodermal activity throughout the relaxation exercise. Guided imagery, *F* = 3.86, *p*=0.008, *η*^2^ = 0.23, and PMR, *F* = 13.40, *p* < 0.001, *η*^2^ = 0.51, groups showed linear decreases in electrodermal activity throughout the relaxation exercise. The deep breathing, *F* = 7.46, *p* < 0.001, *η*^2^ = 0.37, group showed a curvilinear trend where levels of electrodermal activity initially increased in the first 15 minutes and then decreased over the last 10 minutes. Changes in levels of heart rate activity across the four groups only approached statistical significance, *F* = 1.65, *p*=0.084, *η*^2^ = 0.11.

## 4. Discussion

Findings from the present study show effectiveness of all three stress relaxation exercises in promoting both psychological and physiological relaxation states. In terms of psychological relaxation, all groups started at a similar level of relaxation, but following relaxation training, all three stress relaxation groups showed statistically significant increases in relaxation states. Levels of relaxation following the stress relaxation exercises were significantly higher for the relaxation groups as compared to the control group. After the exercises, none of the relaxation groups differed from one another. In terms of physiological states of relaxation, we found that guided imagery and PMR showed linear decreases in phasic electrodermal activity. The deep breathing group showed a curvilinear trend where levels of electrodermal activity increased in the first 10 minutes but in the final 10 minutes returned to their initial levels. Electrodermal activity did not change significantly in the control group, and no statistically significant or interpretable trends occurred in heart rate over the stress relaxation exercises or in the control condition.

These findings support and extend an existing body of findings showing that stress relaxation techniques can be effective in helping individuals to cope with stress and enhance well-being. For instance, with regard to PMR, our findings extend recent work showing that not only does PMR alleviate stress [8] and test anxiety [6] in nursing students and depression and anxiety in coronary heart disease [9] and cancer patients, [11] but it also induced psychological and physiological relaxation states in our student sample. Similarly, our guided imagery technique showed effectiveness in inducing states of both psychological and physiological relaxation, similar to the effects of guided imagery for surgery patients [19, 20], hospital nurses working during the COVID-19 pandemic [17], and healthy adults [18]. Of note is that the work of Felix and Ferreira [20] is a rare recent example of research that confirms the physiological effects of guided imagery, and we extend this finding from a patient sample to our sample of healthy transitional adults.

Our findings on the effectiveness of deep breathing for bringing about psychological relaxation are in line with other studies showing deep breathing to positively impact negative effect, stress, and cortisol [14], studies in medical patients showing decreases in anxiety for surgery patients [19, 20], and individuals with chronic illnesses (e.g., diabetes) [16]. Interestingly, in work by Ma and colleagues [14], deep breathing yielded a net benefit for reduced cortisol levels, while our findings revealed a curvilinear trend—as the deep breathing session progressed, electrodermal activity increased until the approximate midpoint of the training exercise and then began to decrease. Perhaps, early in a training session, deep breathing has an immediate physiological stimulating effect which quickly gives way to a state of physiological relaxation felt in both the autonomic and neuroendocrine systems [28]. As some have argued, focusing too much on the inhalation of deep breathing could cause physiological arousal and, early in training, participants may not understand how to obtain maximal relaxation benefits from deep breathing where one focuses more on the exhalation, which invokes the parasympathetic nervous system [29].

Our study is one of only a few investigations that evaluates the effectiveness of different types of stress relaxation training against each other in a head-to-head comparison, whereas past studies have often investigated combined methods of stress relaxation. For example, in a study of nursing students by Moore and colleagues [22], guided imagery, progressive muscle relaxation, deep breathing exercises, mindfulness, exercise, aromatherapy, and yoga were utilized concurrently. Although the combined effects of multiple stress relaxation techniques may act in synergistic ways to bring about considerable additional relief above and beyond any single method, such studies are unable to isolate which contributing component is responsible for the effect. Other studies have been designed to directly compare the effectiveness of different stress relaxation techniques but, often, these studies [23, 24] have examined unique populations (i.e., elderly oncology patients, pregnant women) or have not found consistent evidence of differences between stress relaxation techniques [7].

Our study offers a direct comparison of three of the more commonly used stress relaxation techniques, finding that all three techniques are more effective in inducing relaxation than the control condition, but none of the three techniques is any better than the others. This holds true for psychological relaxation states but not for physiological states of relaxation, which were evoked more robustly by PMR and guided imagery. For deep breathing, we found an initial increase in physiological arousal, followed by a return to baseline levels, a pattern which may have emerged due to a limited follow-up period. Although it is conjecture, it may be that deep breathing requires a longer period of time to exert its benefits, and its effects may be comparable to PMR and guided imagery with a longer follow-up period. Yet, given the techniques we utilized and the time allotted, it appears that physiological relaxation may be best facilitated by PMR and guided imagery.

### 4.1. Limitations

Our novel findings must be considered in the context of some limitations, including our method of assessing sociodemographic characteristics. For instance, biological sex was measured with only “male” and “female” response options and although our college student population is quite homogeneous (e.g., 85% White, 75% on-campus work-study, no children), no data on employment, parental status, race, or income was collected. Next, although our study design offers the ability to draw causal conclusions about the effectiveness of stress relaxation techniques, only three techniques are evaluated, and many other stress-reduction strategies may be effective. For instance, mindfulness, autogenic training, and aromatherapy are common methods that have received enthusiastic support from the public and should be evaluated in head-to-head comparisons to assess effectiveness.

Although our study goes beyond many studies by including both psychological and physiological assessments, these could be expanded upon in future research. Measures of awe, contentment, and serenity and perceptions of space, self, and time could be added to psychological assessment, and measures of neuroendocrine functioning would be useful. It may be important to also assess neurophysiological functioning with fMRI, PET, or EEG technologies. Finally, while many studies of stress relaxation are conducted with objectively stressed populations (e.g., persons with chronic illness), our study aim was to examine whether stress relaxation methods could be effectively used to reduce stress in healthy, young college students. Yet, this approach also limits generalizability and additional work is needed to substantiate our findings in a diverse community and clinical samples.

## 5. Conclusions

Our study provides evidence of the effectiveness of three commonly used approaches—PMR, deep breathing, and guided imagery—for stress relaxation, confirming past research indicating their benefits for promoting both psychological and physiological states of relaxation and offering a head-to-head comparison of stress-reduction strategies. At the psychological level of analysis, our results suggest that PMR, deep breathing, and guided imagery offer good improvement in relaxation. At the physiological level of analysis, PMR and guided imagery offer good improvement in relaxation, but further work is needed to establish the benefits of deep breathing.

Several avenues of future work are possible, including examination of the effectiveness of relaxation strategies in (1) undergraduate versus graduate student populations, (2) minority groups, (3) the unemployed, and (4) groups with health disparities or poor access to healthcare. Relaxation interventions could also be implemented as a public health effort, to maximize relief at a population level. Future studies might also examine the curvilinear effect of deep breathing on physiological relaxation, using an extended follow-up period and additional measures (e.g., neuroendocrine assessment), to better understand both the immediacy and endurance of the effects of deep breathing on relaxation states. Initial arousal may be viewed as a means to an end if deep breathing proves to have lasting physiological benefits, whereas if the physiological relaxation benefits of deep breathing are fleeting and cyclic in nature, there may be a reason for greater scrutiny. Answering these questions will require longer time intervals of assessment, multimodal physiological assessment, and careful attention to linear and curvilinear patterns of response. Future research comparing stress-reduction strategies utilizing enhanced assessment and diverse samples is needed to better understand what stress relaxation methods bring about the most comprehensive and effective relief of the stresses that impact us.

## Data Availability

Data are available upon request to the first author.
